# Significant Lowering Optical Loss of Electrodes via using Conjugated Polyelectrolytes Interlayer for Organic Laser in Electrically Driven Device Configuration

**DOI:** 10.1038/srep25810

**Published:** 2016-05-11

**Authors:** Jianpeng Yi, Qiaoli Niu, Weidong Xu, Lin Hao, Lei Yang, Lang Chi, Yueting Fang, Jinjin Huang, Ruidong Xia

**Affiliations:** 1 Key Laboratory for Organic Electronics & Information Displays (KLOEID) & Institute of Advanced Materials (IAM), Jiangsu National Synergistic Innovation Center for Advanced Materials (SICAM), Nanjing University of Posts and Telecommunications, 9 Wenyuan Road, Nanjing 210023, China

## Abstract

One of the challenges toward electrically driven organic lasers is the huge optical loss associated with the contact of electrodes and organic gain medium in device. We demonstrated a significant reduction of the optical loss by using our newly developed conjugated polyelectrolytes (CPE) PPFN^+^Br^−^ as interlayer between gain medium and electrode. The optically pumped amplified spontaneous emission (ASE) was observed at very low threshold for PFO as optical gain medium and up to 37 nm thick CPE as interlayer in device configuration, c.f., a 5.7-fold ASE threshold reduction from pump energy 150 μJ/cm^2^ for ITO/PFO to 26.3 μJ/cm^2^ for ITO/PPFN^+^Br^−^/PFO. Furthermore, ASE narrowing displayed at pump energy up to 61.8 μJ/cm^2^ for device ITO/PEDOT:PSS/PFO/PPFN^+^Br^−^/Ag, while no ASE was observed for the reference devices without CPE interlayer at pump energy up to 240 μJ/cm^2^. The optically pumped lasing operation has also been achieved at threshold up to 45 μJ/cm^2^ for one-dimensional distributed feedback laser fabricated on ITO etched grating in devices with CPE interlayer, demonstrating a promising device configuration for addressing the challenge of electrically driven organic lasers.

Although optically pumped organic semiconductor lasers have been demonstrated with high slope efficiency and broad lasing wavelength on various feedback structures since 1996^1^,^2^,^3^,^4^,^5^,^6^, electrically driven organic lasers still remain a great challenge. Two major obstacles have to be overcome in the pursuit of electrically driven organic lasers. First, low carrier mobility of the organic materials make it impossible to generate high current density required for the lasing actions. Second, the huge optical losses associated with the contact of electrodes and organic gain media is detrimental for the amplified spontaneous emission (ASE) action. Efforts including designation of materials and modifications of traditional device configuration have been paid to address these issues^7^,^8^,^9^,^10^,^11^. The demonstration of copolymers combing excellent optical gain property and high charge carrier mobility brings the organic laser research one step forward to achieve the electrically-driven device^2^,^12^. However, huge threshold increase or eventually quenching ASE is commonly observed in the devices of gain media contacted with electrodes. This problem has to be addressed in device configuration. Although thermally evaporated or sputtered SiO_2_ layers with about 100 nm thickness have been introduced between electrode and gain media to reduce the losses associated with the electrode contact^13^,^14^,^15^, SiO_2_ could also block electrons and holes injecting from electrode into the active layer due to its insulation character. As a result, the device with SiO_2_ as interlayer is hardly to reach the high current density required by laser action. Therefore, a conductive interlayer combining simple and convenient fabrication approaches is highly desirable for electrically driven organic laser device.

In recent years, conjugated polyelectrolytes (CPEs) have been employed to modify the energy level of the indium-tinoxide (ITO) or metal electrode, therefore, to improve electron injection/extraction ability for organic light emitting devices (OLEDs) and organic solar cells (OSCs)^16^,^17^,^18^,^19^,^20^,^21^. The thin CPE layers (up to 30 nm) with good conductivity make them are excellent hole or electronic transport layers in either conventional devices or inverted devices. Good solubility of CPEs in polar solvents such as water and alcohol offers the possibility to avoid interfacial mixing during the solution-based fabrication of multilayer. However, the use of CPE interlayer for reducing optical loss by ITO or metal electrode in organic laser device has not been explored yet. In this work, we developed a novel polyfluorene derivatives poly [9,9-bis(4′-(6′-(N,N,N-trimethylammonione) hexyloxy)phenyl)fluorene] bromide (PPFN^+^Br^−^), which shows improved morphology and higher refractive index with respect to its counterpart 9,9′-bis[69-(N,N,N- trimethylammonium) hexyl] (PFNBr)^17^,^19^. We have studied the ASE threshold in detail at various device configurations with PPFN^+^Br^−^, PFNBr and Poly (3,4-ethylenedioxythiophene)/poly (styrenesulfonate) (PEDOT:PSS) as interlayers between electrodes and gain medium. The laser gain medium used in our study is blue-emitting conjugated polymer poly(9,9-dioctylfluorene) (PFO)^2^,^22^. The chemical structures, normalized absorption and PL of PFO, PFNBr, PPFN^+^Br and PEDOT:PSS are shown in Supplementary Information (Figs S1 and S2 in SI). We observed ASE started at very low threshold from the device of glass/ITO /interlayer/PFO by optimizing the thickness of interlayer films. Furthermore, PPFN^+^Br^−^ was used as interlayer between metal electrode and PFO since it works better than PFN^+^Br and PEDOT:PSS. Our study shows the PPFN^+^Br^−^ has blocked light quenching of the metal electrode efficiently, therefore ASE of PFO was observed from device ITO/PEDOT:PSS/PFO/PPFN^+^Br^−^/Ag, while no ASE was observed for the reference devices without CPE interlayer at pump energy up to 240 μJ/cm^2^. Following detailed study of ASE threshold on various device configurations, the optically pumped lasing operation has also been demonstrated for one-dimensional (1-D) distributed feedback (DFB) laser fabricated on ITO etched grating in a typical device configuration with CPE interlayer. The results suggest introducing CPE interlayer could be a promising approach to address the optical loss of electrode in electrically driven organic laser device.

## Results

**Figure 1:**
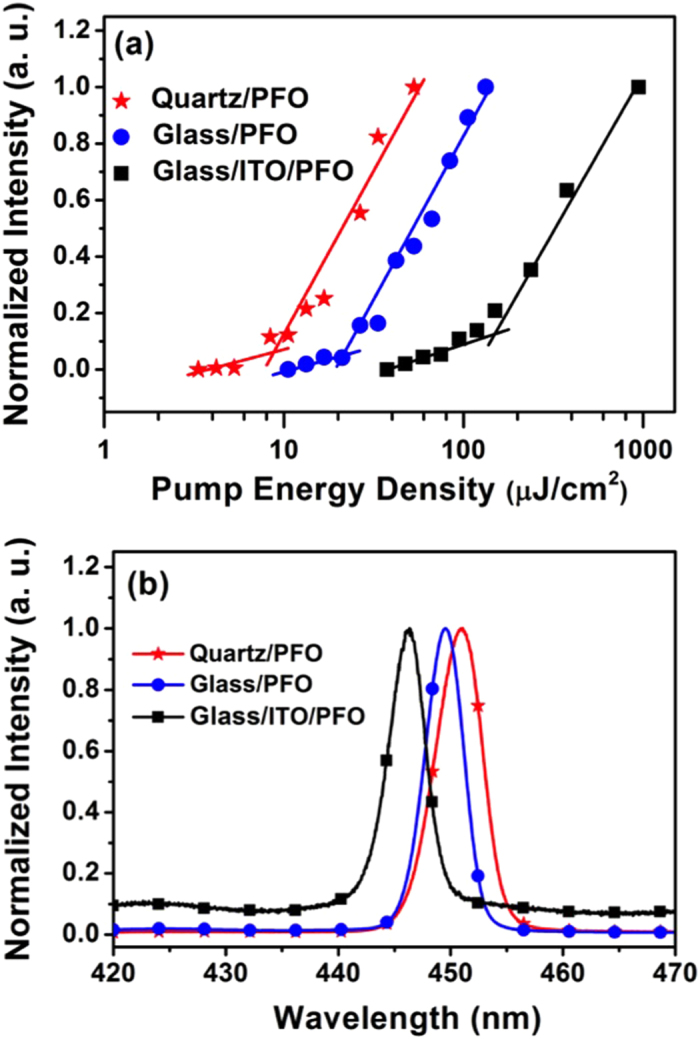
(**a**) The normalized output intensity as a function of the pump energy density and (**b**) the normalized ASE spectra at pumping energy well above the threshold for PFO (90 ± 2 nm) slab waveguide based on various substrates.

The ASE threshold of PFO as a function of film thickness was first investigated by depositing PFO film on quartz, glass and ITO (120 nm) coated glass as substrates (see Figs S3 and S4 in SI). Figure 1 shows the output ASE intensity versus the pump energy density and the ASE spectra at pumping energy well above the threshold for the quartz/PFO, glass/PFO, glass/ITO/PFO planar waveguides at same PFO film thickness of 90 nm. The lines in Fig. 1a represent the linear fits to experimental data in the two linear regimes of spontaneous emission (SE) and amplified spontaneous emission (ASE). With the increased pump energy density, a transition of the slope exists in the fit lines indicating the onset of ASE, where the full width at half maximum (FWHM) of the emission spectrum halves. We define the pump energy density at this point as ASE threshold. When PFO deposited on quartz, the lowest ASE threshold of 8.4 μJ/cm^2^ was observed at film thickness of 90 ± 2 nm (see Fig. S3a). The ASE peak wavelength was at 451 nm with FWHM of 4.6 nm. The ASE spectral window was 19 nm from 443–462 nm by increasing the film thickness from 38–185 nm (see Fig. S3b). These characterization results are comparable with values reported previously,^22^ indicating that the product is of good quality to support the present work. However, as shown in Fig. 1a, the ASE threshold was 20.6 μJ/cm^2^ for the glass/PFO, 2.5 fold increase respect to that of quartz/PFO, and 150 μJ/cm^2^ for glass/ITO/PFO, 9 fold higher than the ASE threshold of quartz/PFO. The thickness of PFO film has to be increased to 150 nm to reduce the ASE threshold down to the lowest value of 50 μJ/cm^2^ for ITO/PFO (Fig. S4a in SI). Nevertheless, it was still 6 fold higher than ASE threshold of quartz/PFO, while the thick film and low mobility of PFO will make it extremely difficulty to achieve the high current density desired for lasing in electrically driven device.

We noted the low ASE threshold of quartz/PFO could be ascribed to good optical confinement due to the relatively low refractive index of quartz respect to the index of PFO. The refractive index (n) at wavelength of 450 nm is 1.76 for PFO, 1.46 for quartz, 1.53 for glass and 1.9 for ITO. The relatively high index of ITO lead to high optical leakage loss between PFO and ITO interface, i.e., bad optical confinement^2^,^13^,^14^, therefore, high ASE threshold. On the other hand, the extremely rough surface of ITO causes strong scattering loss^18^,^21^. This is another reason resulting in the highest ASE threshold of glass/ITO/PFO among the three devices. As a matter of fact, traditional electrodes, like ITO, would severely cripple ASE action of gain media, which is one of the obstacles for realizing electrically driven organic lasers. Therefore, suitable interlayer between ITO and active gain medium has to be developed to reduce the ASE threshold while allowing high density of current injection through electrode to gain media.

**Figure 2:**
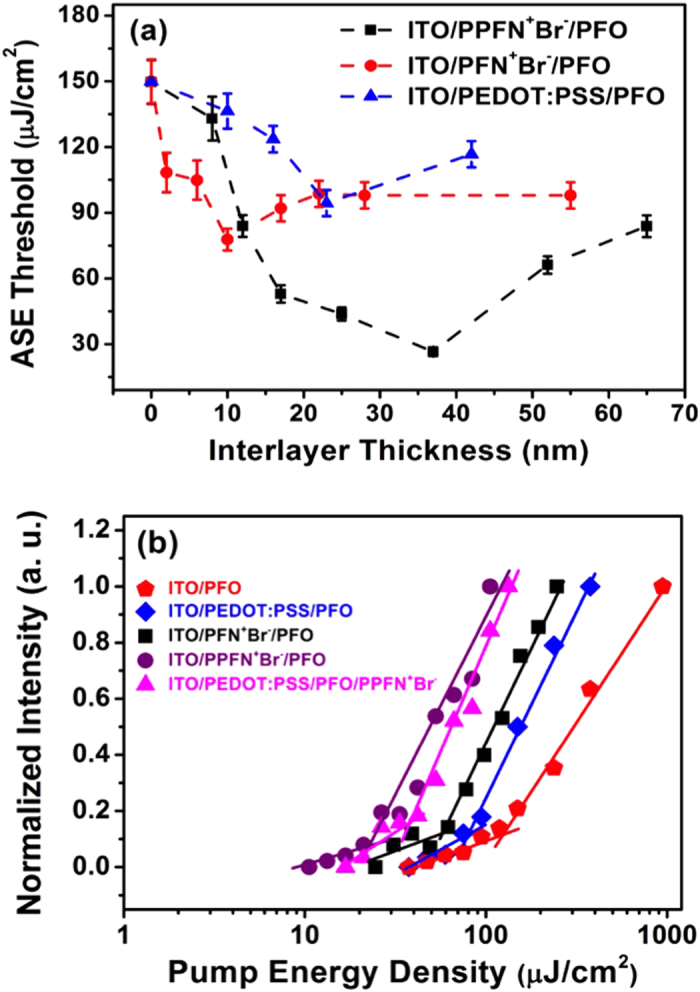
(**a**) The ASE threshold of PFO as a function of the thickness of interlayers. (**b**) The normalized output intensity of PFO emission versus the pump energy density for various device configurations at optimized interlayer thickness.

PFN^+^Br^−^, PPFN^+^Br^−^ and PEDOT:PSS were investigated as the interlayers to modify the surface of ITO in pursuit of ASE threshold reduction in the device configuration of ITO/interlayer/PFO. The thickness of PFO is fixed at 90 nm. The thickness of interlayers was controlled by solution concentration and spin-coating speed. It was calculated by Beer’s law (see Fig. S5 in SI). The optimization procedure of the interlayers for each device is shown in Fig. S6 in SI. Figure 2a shows the ASE threshold as a function of interlayer thickness for devices ITO/interlayer/PFO. The ASE threshold of the PEDOT:PSS-based device decreases from 150 μJ/cm^2^ to 94.4 μJ/cm^2^ (1.6-fold reduction) with the PEDOT:PSS thickness increased to 20 nm, then started to increase as the PEDOT:PSS thickness further increase. Similar trend was observed for PFN^+^Br^−^-based device, i.e., a gradual reduction of the ASE threshold with the PFN^+^Br^−^ thickness increasing up to 13 nm, where the lowest threshold of 62.2 μJ/cm^2^ (2.4-fold reduction) emerges. Further increase of the PFN^+^Br^−^ thickness did not contribute to the reduction of the ASE threshold. As for the PPFN^+^Br^−^-based devices, with the PPFN^+^Br^−^ thickness increased to 37 nm, the ASE threshold quickly decreased and the lowest threshold of 26.3 μJ/cm^2^ (5.7-fold reduction) was obtained, which is comparable with the ASE threshold of glass/PFO although it was still higher than that of quartz/PFO. We note that further increasing the thickness of interlayer beyond the optimized thickness lead to ASE threshold increase for all the three interlayers. This could be caused by the absorption of the interlayer (see Fig. S2 in SI) and/or the film quality of the interlayer became poor. Figure 2b shows the output intensity versus the pump energy density for various device configurations at optimized thickness of each interlayer.

**Figure 3:**
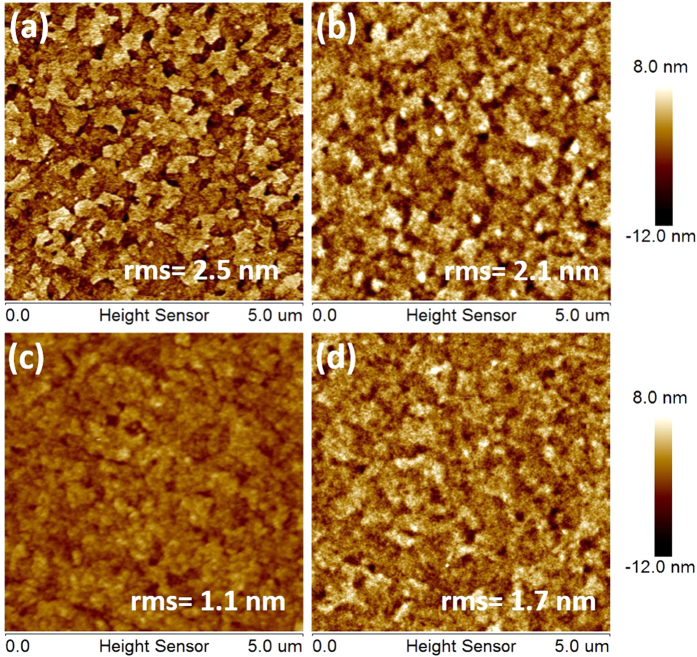
AFM images of (**a**) bare ITO, (**b**) ITO/PFN^+^Br^−^(14 nm), (**c**) ITO/ PPFN^+^Br^−^ (37 nm) and (**d**) ITO/ PEDOT:PSS (23 nm).

The surface morphology of the devices is believed to be one of the most important elements in optical waveguide. We therefore investigated the morphology of bare ITO and ITO/interlayer by means of atomic force microscope (AFM). Figure 3 is the AFM images of bare ITO and ITO coated with three interlayers at the thickness on which the lowest ASE threshold was achieved. The root mean square roughness (RMS) is 2.5 nm for bare ITO, 2.1 nm for ITO/ PFN^+^Br^−^ (14 nm), 1.7 nm for ITO/PEDOT:PSS (23 nm) and 1.1 nm for ITO//PPFN^+^Br^−^ (37 nm). The AFM images clearly show the surface roughness of ITO has been improved by insertion of interlayer. The improved surface morphology certainly reduced the light scattering loss, therefore, lowering the ASE threshold. The most significant improvement of ITO surface morphology was achieved by PPFN^+^Br^−^ as interlayer. We note the RMS of 23 nm PEDOT:PSS coated ITO is smaller than 14 nm PFN^+^Br^−^ coated one. However, the device with PEDOT:PSS interlayer exhibited higher ASE threshold with respect to the ASE threshold of the device with PFN^+^Br^−^ interlayer as shown in Fig. 2a,. One of the reason could be that PEDOT:PSS layer would quench the excitons generated by the gain medium under optically-pumped conditions, therefore, impede the ASE action. Similar phenomenon has been reported in literatures on OLEDs^23^,^24^,^25^.

To better understand the optical confinement of the interlayer incorporated device, we measured the refractive index of the interlayers (see Fig. S7 in SI). The indexes at ASE wavelength of PFO (450 nm) are 1.62 for PEDOT:PSS, 1.70 for PFN^+^Br^−^, and 1.74 for PPFN^+^Br^−^. They are all lower than the index of ITO (n = 1.90). Therefore, the optical leakage at interlayer/ ITO interface could not be completely avoided by inserting the interlayer between PFO and ITO. However, relatively high index of interlayer is obviously beneficial for optical confinement within gain medium, therefore, lowering ASE threshold. Among the three interlayers used here, PPFN^+^Br^−^ caused the most significant reduction of the ASE threshold due to improved morphology and its highest refractive index. Taking all the results into account, we conclude that uniform surface morphology, less exciton quench and high refractive index of the interlayer all contributed to reduce ASE thresholds. Among the three factors improved surface morphology, which lead to less light scattering loss, and reduced exciton quench are more important than index increase at the interlayer thickness up to 40 nm as we used since the waveguide modes confinement may not change significantly at such thin layer^14^.

**Figure 4:**
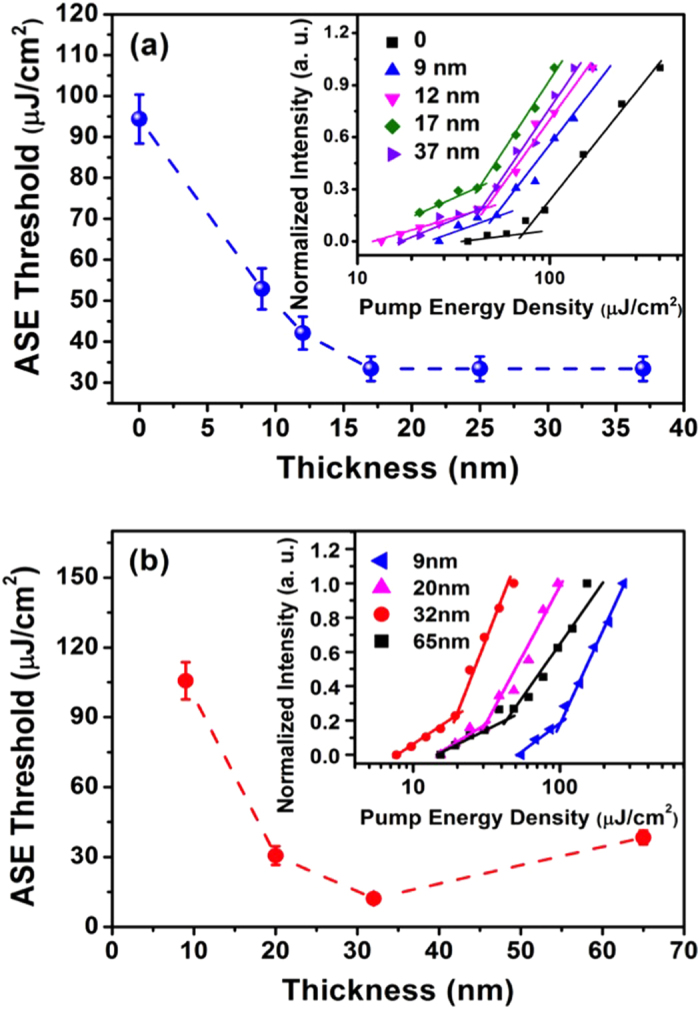
The ASE threshold as a function of the thickness of PPFN^+^Br^−^ film based on the device structure of (**a**) glass/ITO/PEDOT:PSS (23 nm)/PFO (90 nm)/PPFN^+^Br^−^ and (**b**) glass/Ag (40 nm)/PPFN^+^Br^−^/PFO (90 nm). The inset of (**a**) and (**b**): normalized intensity as a function of the pump energy density with various film thicknesses of PPFN^+^Br^−^.

Another major loss in device structure is the absorption quench of the metal electrode to exciton (i.e. metal quench effect)^14^,^15^. It is well known that metal surface would seriously quench the excitons generated in the active media under the external excitation conditions including optically pumping and electrically driving. Therefore, the second part of this work was investigating the performance of water/alcohol soluble CPEs as interlayer between gain medium and metal electrode on reducing metal quench effect. For a detailed study, we tested PPFN^+^Br^−^ as interlayer between metal electrode and PFO. We started with investigating the ASE performance as a function of the thickness of top PPFN^+^Br^−^ layer for device glass/ITO/PEDOT:PSS (23 nm)/PFO (90 nm)/ PPFN^+^Br^−^. The device was pumped from ITO glass side. Figure 4a shows the ASE threshold at various thickness of PPFN^+^Br^−^ layer. The device with 17 nm top PPFN^+^Br^−^ layer (glass/ITO/PEDOT:PSS/PFO/PPFN^+^Br^−^) demonstrated an impressively low ASE threshold (33.4 μJ/cm^2^, 2.8-fold decrease, see also Fig. S8a in SI) with respect to the asymmetric waveguide of glass/ITO/PEDOT:PSS/PFO/air (94.4 μJ/cm^2^ in Fig. 2a).

Furthermore, to exam the influence of metal electrode quenching effect on the ASE action of the gain medium, several devices with Ag electrode (40 nm thickness) were fabricated as glass/Ag/PFO, glass/ITO/PFO/Ag, and glass/ITO/PEDOT:PSS/PFO/Ag. The morphology of the Ag and Ag/PFO films was reasonable uniform (see Fig. S9). However, none of the devices show clear ASE narrowing at pump energy density up to 240 μJ/cm^2^ due to the severe excitons quenching effect of the metal electrode. We therefore introduced PPFN^+^Br^−^ as interlayer between Ag electrode and PFO to fabricate device glass/Ag/PPFN^+^Br^−^/PFO. Figure 4b shows the ASE threshold of PFO as a function of the thickness of PPFN^+^Br^−^ film of this device. The ASE of PFO started to emerge at the PPFN^+^Br^−^ layer up to 9 nm. The ASE threshold decreased continuously with the thickness of PPFN^+^Br^−^ increasing up to 32 nm and then started to go up with thickness of PPFN^+^Br^−^ further increases. The lowest ASE threshold was 13.3 μJ/cm^2^ at the PPFN^+^Br^−^ thickness of 32 nm (see also Fig. S8b in SI). Following these two tests, we fabricated device glass/ITO/PEDOT:PSS (23 nm)/PFO (90 nm)/PPFN^+^Br^−^ (22 nm)/Ag (100 nm). A clear ASE narrow spectrum appeared at pump energy up to 61.8 μJ/cm^2^ (1.12 μJ/pulse) with the peak wavelength at 447 nm and the FWHM of 3.8 nm. These tests confirmed that inserting PPFN^+^Br^−^ between PFO and Ag was an efficient approach on reducing the exciton losses caused by quenching effect of the Ag electrode.

**Figure 5:**
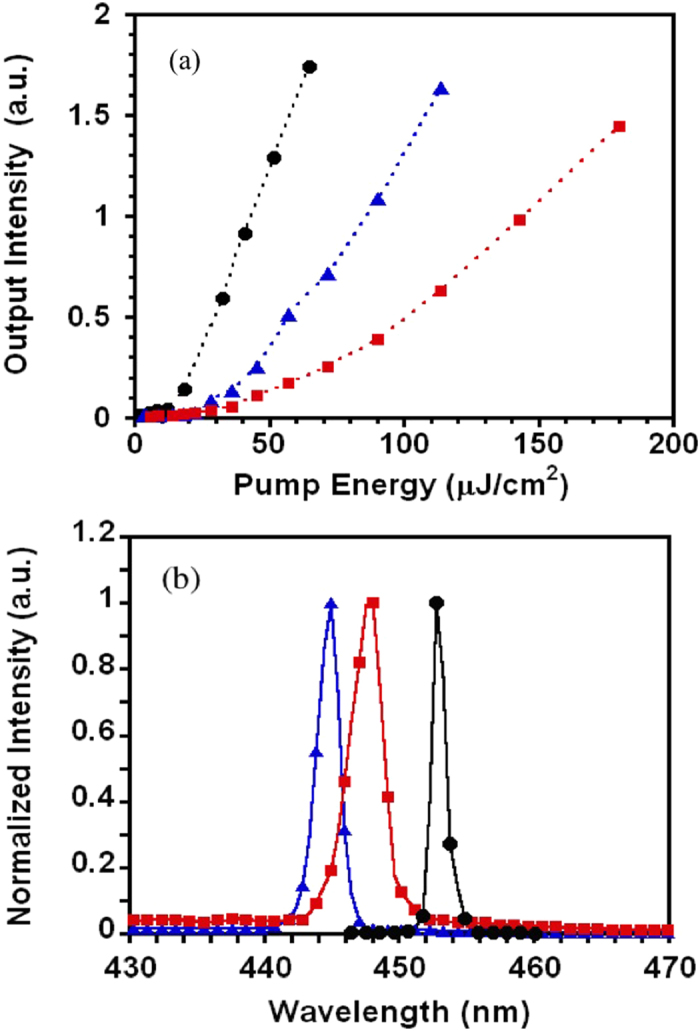
(**a**) Output intensity of 1-D DFB laser (Λ = 290 nm period etched ITO grating, 50% fill factor, 100 nm depth) as a function of pump energy and (**b**) laser emission spectra at pump energy well above the threshold for three devices: glass/ITO grating/PPFN^+^Br^−^ (26 nm) /PFO (98 nm) (filled cycles), glass/ITO grating/PEDOT:PSS (25 nm)/PFO (88 nm)/PPFN^+^Br^−^ (22 nm) (filled triangles) and glass/ITO grating/PEDOT:PSS (25 nm)/PFO (92 nm)/PPFN^+^Br^−^ (22 nm)/Ag (100 nm) (filled squares).

Having observed encouraging ASE results, distributed feedback (DFB) lasers were fabricated by spin coating thin films of the interlayers and gain medium on top of one-dimensional (1-D) gratings etched on ITO glass. The pre-etched ITO comprises parallel 145 nm width, 100 nm depth stripes, each separated by 145 nm (i.e. 290 nm period, 50% fill-factor). The lasing performance of three device configurations was tested under pulsed optical excitation. Figure 5 shows (a) the output intensity at lasing wavelength as a function of pump pulse energy and (b) the laser emission spectra of each device. The laser thresholds and peak wavelengths were 17.8 μJ/cm^2^ and 451 nm for device glass/ITO grating/PPFN^+^Br^−^ (26 nm) /PFO (98 nm), 28 μJ/cm^2^ and 445 nm for device glass/ITO grating/PEDOT:PSS (25 nm)/PFO (88 nm)/PPFN^+^Br^−^ (22 nm) and 45 μJ/cm^2^ and 448 nm for a typical device configuration including metal electrode, glass/ITO grating/PEDOT:PSS (25 nm)/PFO (92 nm)/PPFN^+^Br^−^ (22 nm)/Ag (100 nm). The laser wavelength shift could be due to the thickness change of the interlayer and PFO. These threshold values are much higher than the best values reported for PFO laser fabricated on pre-etched quartz. This is partly due to in that cases more efficient 75% gratings were used rather than the 50% gratings used here^2^. Nevertheless, these results suggest that introducing CPE interlayer between gain medium and electrode could be a promising approach to address the challenge of optical loss of electrode for electrically driven organic laser device.

## Discussion

In conclusion, we presented a systematic investigation of the influence of traditional electrodes (ITO and Ag) on the ASE action of PFO. A significant reduction of the ASE threshold was demonstrated by introducing water/alcohol soluble conjugated polyelectrolytes as interlayer between electrode (ITO and metal) and gain medium. Using PPFN^+^Br^−^ as interlayer, a 5.7-fold reduction of ASE threshold of PFO was observed at glass/ITO/PPFN^+^Br^−^/PFO device with respect to at glass/ITO/PFO device. We attribute this remarkable reduction of ASE threshold to improved surface morphology, less exciton quench and high refractive index of the interlayer. Furthermore, when PPFN^+^Br^−^ inserted between Ag and PFO, ASE was achieved at extremely low pump energy (13.3 μJ/cm^2^). ASE narrowing spectrum was also obtained at pump energy up to 61.8 μJ/cm^2^ for device glass/ITO/PEDOT:PSS/PFO/PPFN^+^Br^−^/Ag, while no ASE was observed for the counterpart devices without CPE interlayer at pump energy up to 240 μJ/cm^2^. Our study shows the newly developed CPE interlayer worked more efficiently in reducing the ASE threshold compared with conventional interlayer PEDOT:PSS. Finally, optically pumped 1-D DFB lasers fabricated on ITO etched grating were demonstrated at lasing threshold up to 45 μJ/cm^2^ in a typical device configuration. The experimental results of ASE and DFB laser study in this work suggest that inducing CPE, such as PPFN^+^Br^−^, as interlayer material between gain medium and electrode could be a promising strategy in the future electrically-pumped organic laser device configuration with considering the excellent performance of CPE on reducing optical loss combined with its previously reported ability on modifying work function of electrode.

## Methods

PFO was purchased from Xi’an Polymer Light Technology and used as received. PEDOT:PSS (Clevios TM Al4083) was used as purchased from Heraeus. The PFN^+^Br^−^ and PPFN^+^Br^−^ were synthesized in our group. All layers except the Ag electrode were deposited by spin-coating in ambient atmosphere except for Ag/PFO and Ag/PFO/interlayer films which was deposited in the glove box with nitrogen atmosphere once the Ag film was thermally evaporated. The Ag layer was prepared by thermally evaporating in vacuum chamber at 1.2 × 10^−4^ Pa. To obtain uniform and smooth Ag film, it was deposited at a constant and moderate speed of 0.1 nm/s. The thickness of the Ag film was ~ 40 nm or 100 nm. The PFO planar waveguides were made by spin-coating 36–180 nm nm thick films from 15, 20, and 30 mg/mL toluene solutions on the precleaned quartz, glass, glass/ITO (120 nm) substrates. DFB lasers were fabricated by pre-patterning ITO substrates with one-dimensional surface grating structures and then spin coating thin film of interlayer and PFO on top. Gratings with a 290 nm period and 145 nm width, 100 nm depth etched stripe (i.e. 50% fill factor) were used.

The UV-Vis absorption and photoluminescence spectra were recorded by Shimadzu UV-3600 spectrophotometer and Shimadzu Luminescence Spectrometer LS 50, respectively. The surface morphology images were taken by Bruker icon Dimension with scanAsyst atomic force microscope (AFM). The film thickness, if over 30 nm, was measured on Bruker DektakXT Stylus Profiling System, otherwise, calculated according to Beer’s law (see Fig. S5 in SI).

For ASE study, the samples were optically pumped by using a Q-switched Nd^3+^:YAG laser pumped optical parametric oscillator (OPO, 10 Hz, 5 ns). The excitation wavelength (370 nm) was close to the maximum absorption peak of PFO. The pump beam was focused on the PFO layer with a rectangle area of 4.1 mm x 440 μm and the emission light was collected from the edge of the film. DFB lasers were optically pumped with the same source, using a 190 μm x 170 μm rectangular spot. The pump energy was adjusted by a set of neutral density filters. The output emission was monitored with an Oriel Instruments fibre-coupled spectrograph equipped with a charge coupled device (CCD) detector.

## Additional Information

**How to cite this article**: Yi, J. *et al*. Significant Lowering Optical Loss of Electrodes via using Conjugated Polyelectrolytes Interlayer for Organic Laser in Electrically Driven Device Configuration. *Sci. Rep.* **6**, 25810; doi: 10.1038/srep25810 (2016).

## Supplementary Material

Supplementary Information
