# Supplementary material for: Significant Lowering Optical Loss of Electrodes via using Conjugated Polyelectrolytes Interlayer for Organic Laser in Electrically Driven Device Configuration
Source: Sci Rep. 2016 May 11;6:25810. doi: 10.1038/srep25810 (PMC4863163; doi:10.1038/srep25810)
Supplement: Supplementary Information [file srep25810-s1.pdf]

Supplementary Information

Significant Lowering Optical Loss of Electrodes via using Conjugated Polyelectrolytes Interlayer for Organic Laser in Electrically Driven Device Configuration

Jianpeng Yi, Qiaoli Niu, Weidong Xu, Lin Hao, Lei Yang, Lang Chi, Yueting Fang, Jinjin Huang, and Ruidong Xia*

Key Laboratory for Organic Electronics and Information Displays & Institute of Advanced Materials (IAM), Jiangsu National Synergistic Innovation Center for Advanced Materials (SICAM), Nanjing University of Posts & Telecommunications, 9 Wenyuan Road, Nanjing 210023, China.

*Corresponding Author: Prof. R. Xia (iamrdxia@njupt.edu.cn)

1. PFO and Interlayer materials

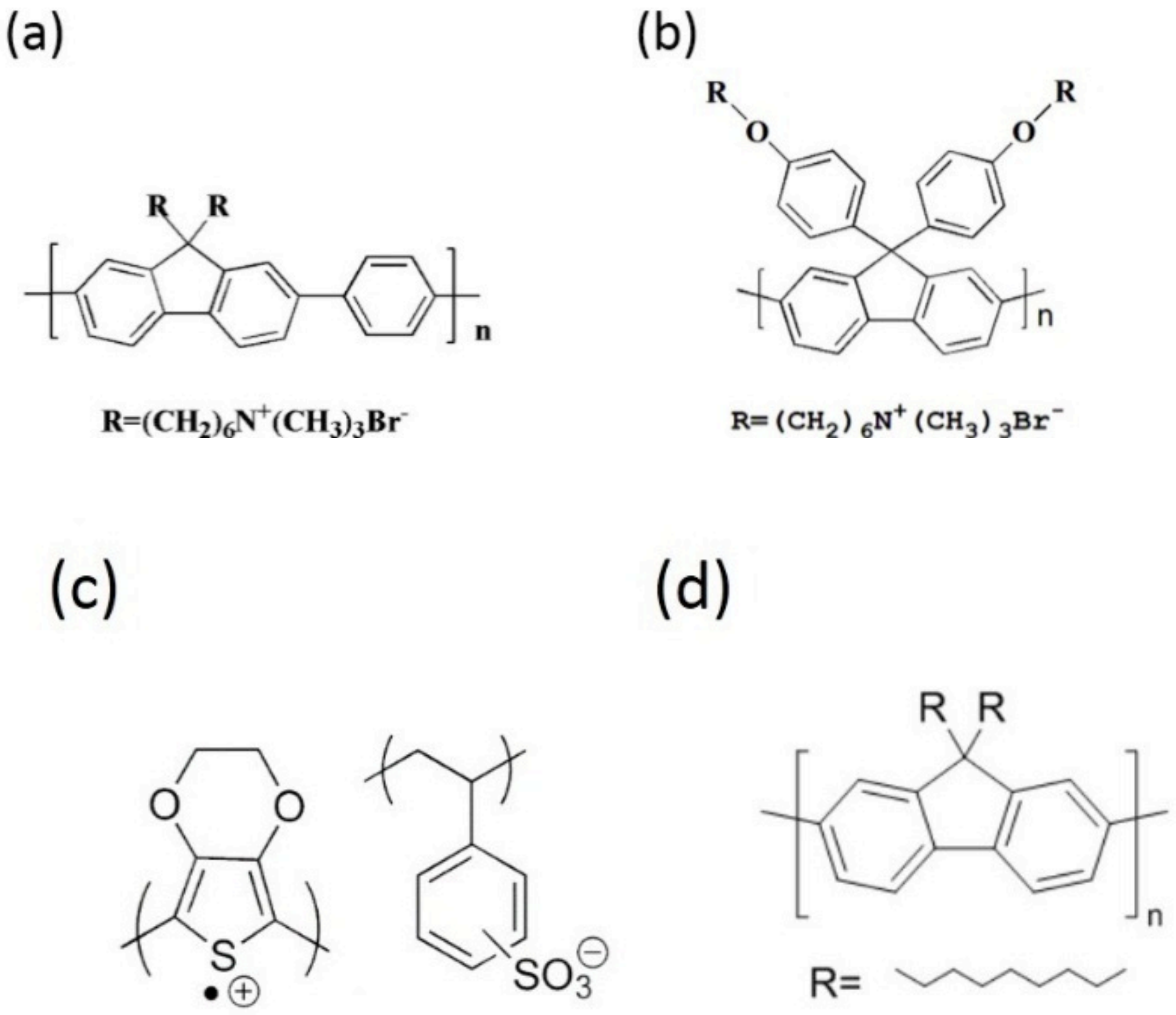

Figure S1. Chemical structures of (a) PFN^+Br^-, (b) $PPFN^+Br^-$, (c) PEDOT:PSS, and (d) PFO.

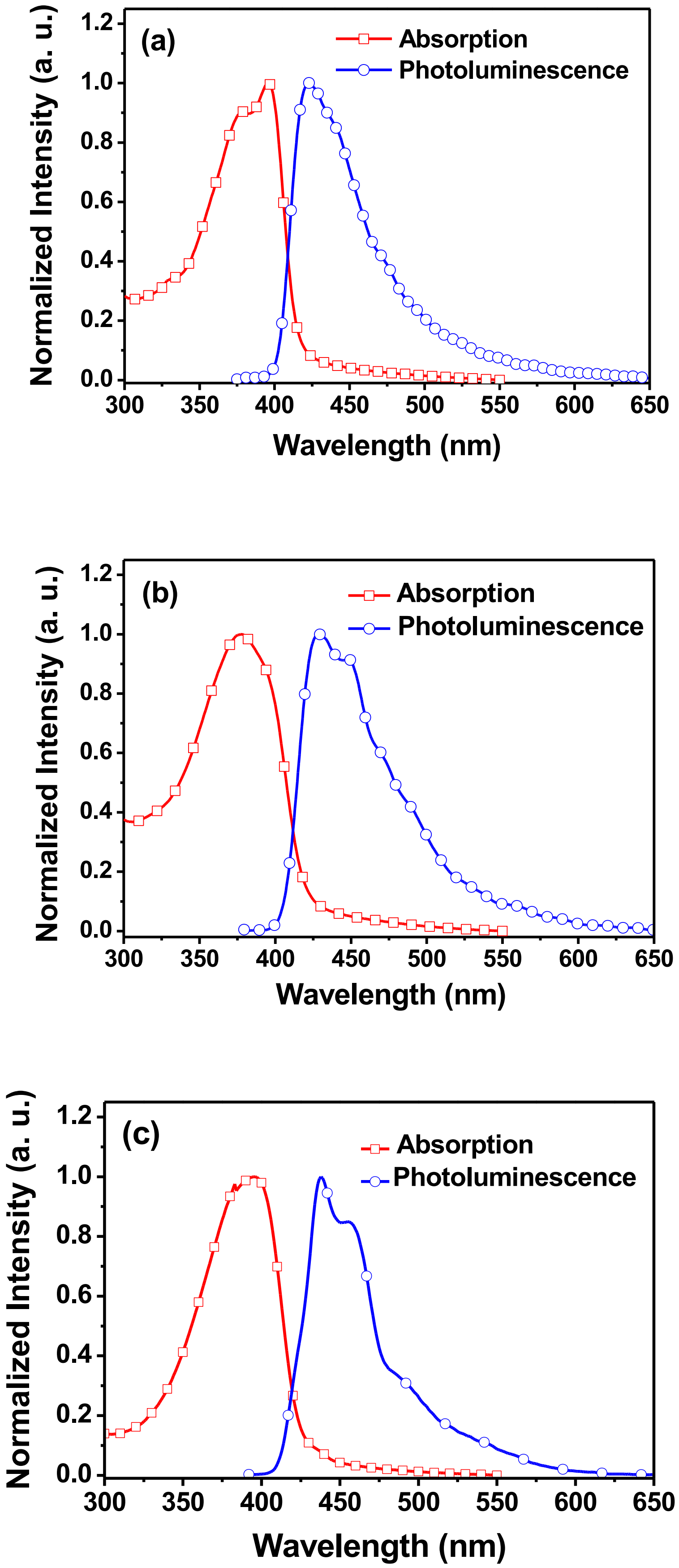

Figure S2. Normalized UV-Vis absorption and photoluminescence spectra of (a) $PFN^{+}Br^{-}$, (b)

PPFN$^+$Br$^-$ and (c) PFO in film.

2. ASE characterizations for PFO deposited at quartz and glass/ITO

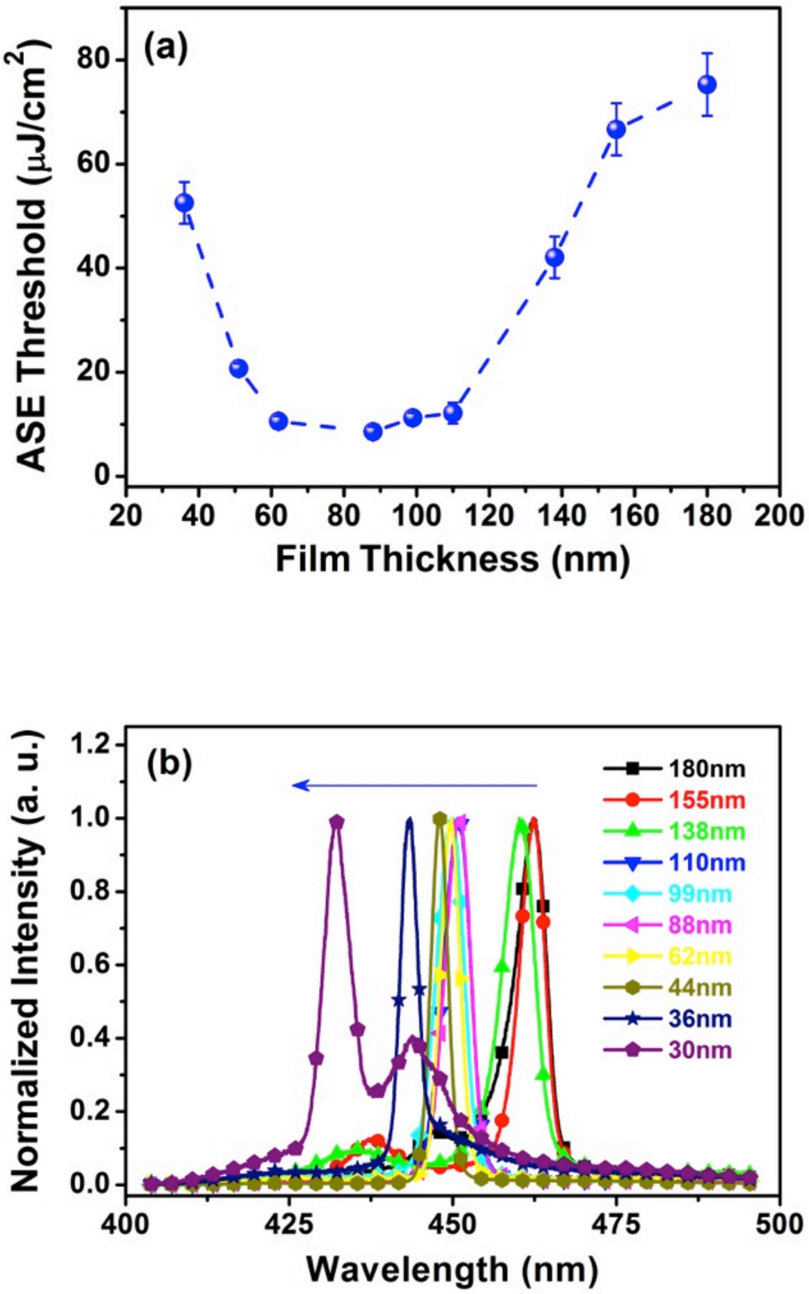

Figure S3. (a) The ASE threshold and (b) the ASE spectra of PFO as a function of the PFO film thickness for quartz/PFO.

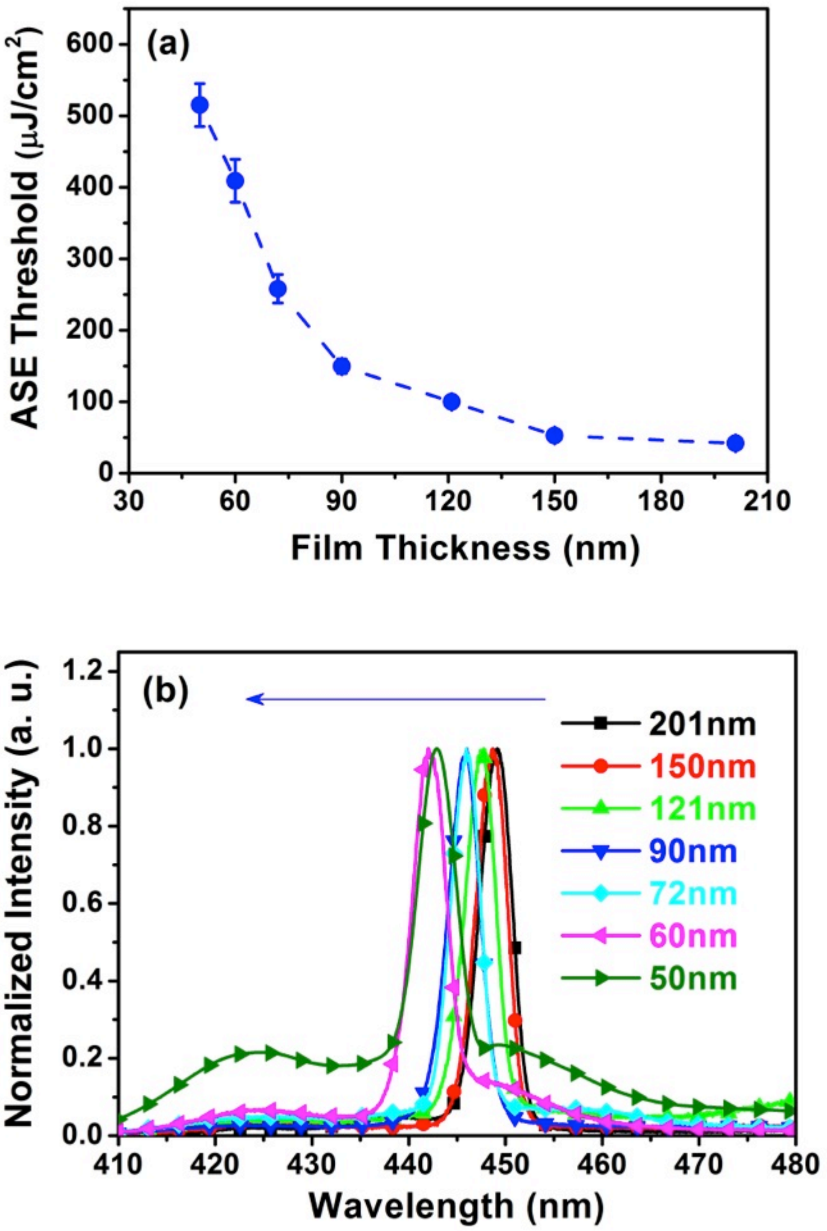

Figure S4. (a) The ASE threshold and (b) the ASE spectra of PFO as a function of the PFO film thickness for glass/ITO/PFO.

2. Determination of the CPE interlayer film thickness

The average film thickness of interlayers was calculated according to Beer's law:

$$\varepsilon = \frac{A}{l}$$

where ε is the extinction coefficients, A is the absorption intensity, l is the film thickness.

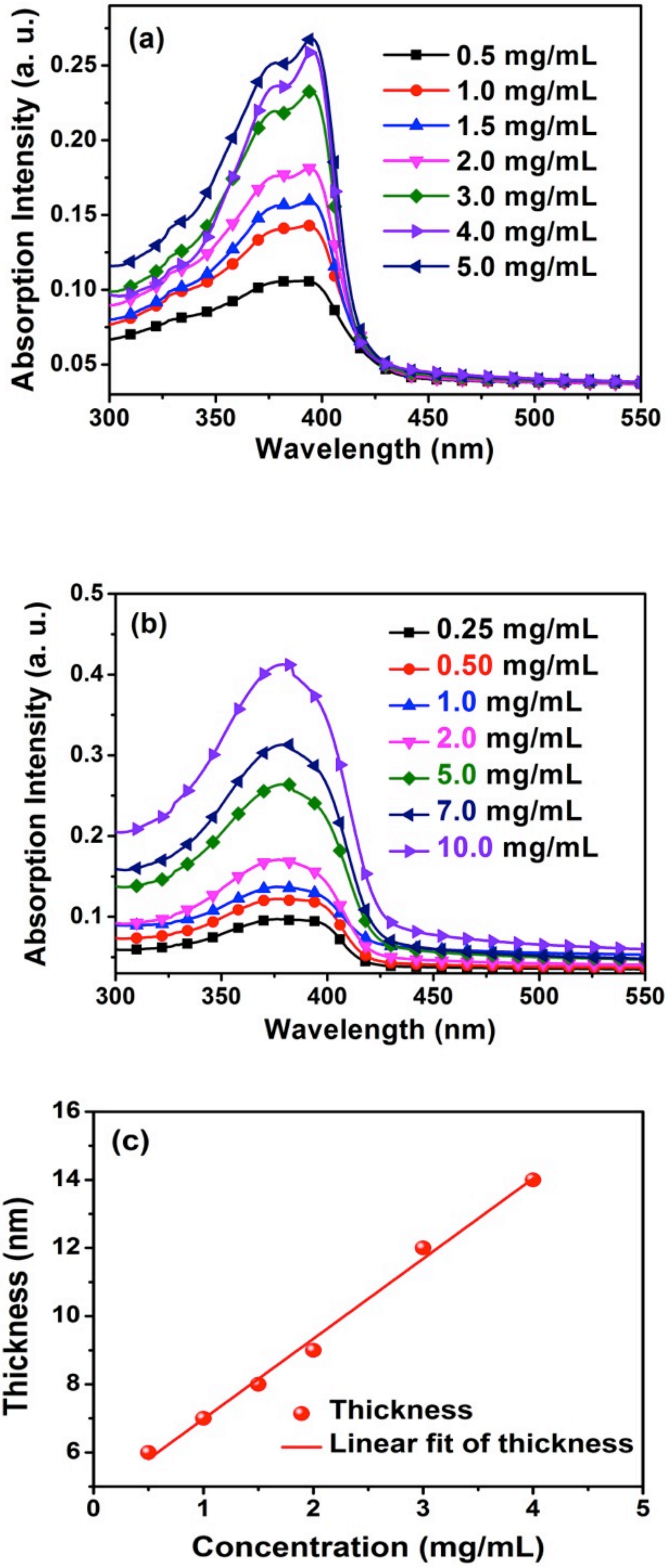
(a)
Absorption Intensity (a. u.)
Wavelength (nm)
0.5 mg/mL
1.0 mg/mL
1.5 mg/mL
2.0 mg/mL
3.0 mg/mL
4.0 mg/mL
5.0 mg/mL
(b)
Absorption Intensity (a. u.)
Wavelength (nm)
0.25 mg/mL
0.50 mg/mL
1.0 mg/mL
2.0 mg/mL
5.0 mg/mL
7.0 mg/mL
10.0 mg/mL
(c)
Thickness (nm)
Concentration (mg/mL)
Thickness
Linear fit of thickness

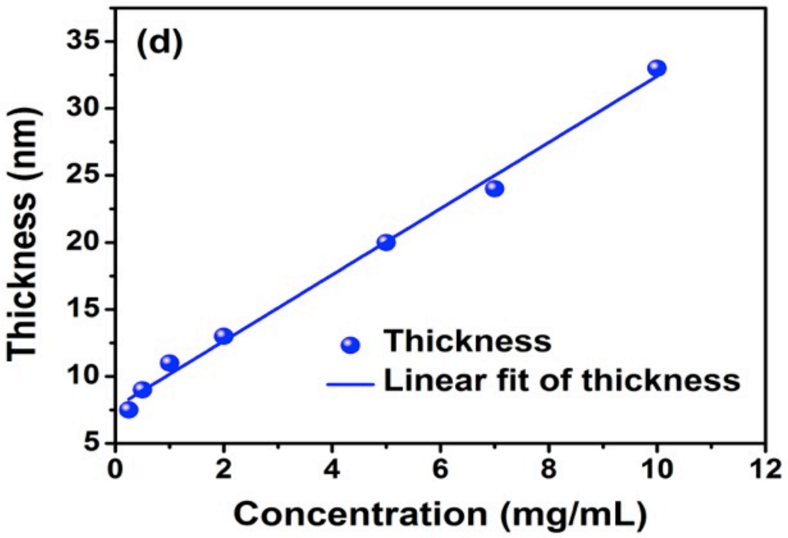

Figure S5. UV-Vis absorption spectra of films spin-coated from various concentration of (a) PFN^+Br^- and (b) $PPFN^+Br^-$ solution at spinning speed of 2000 rpm. The calculated film thickness as a function of solution concentration of (c) PFN^+Br^- and (d) $PPFN^+Br^-$ with fixed spinning speed of 2000 rpm.

4. ASE characterizations for device configuration of ITO/interlayer/PFO

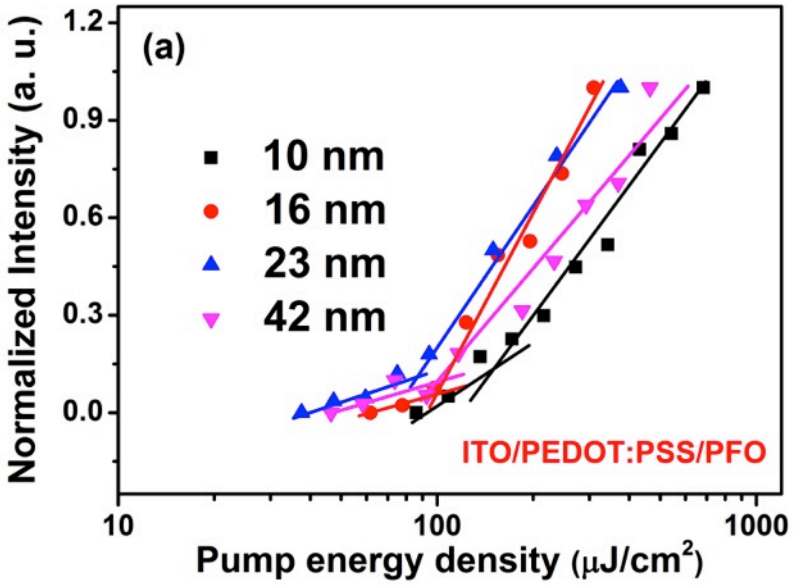

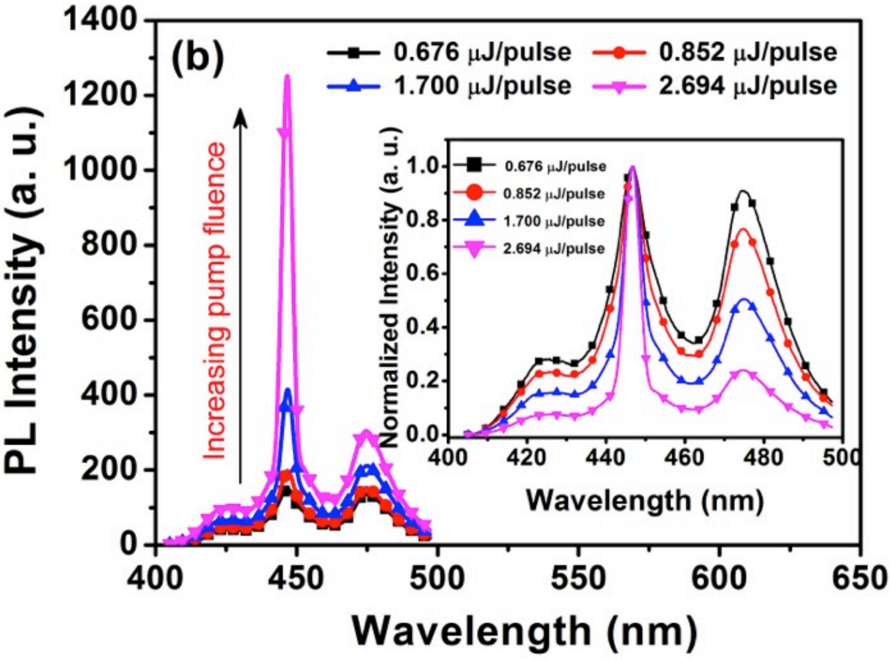

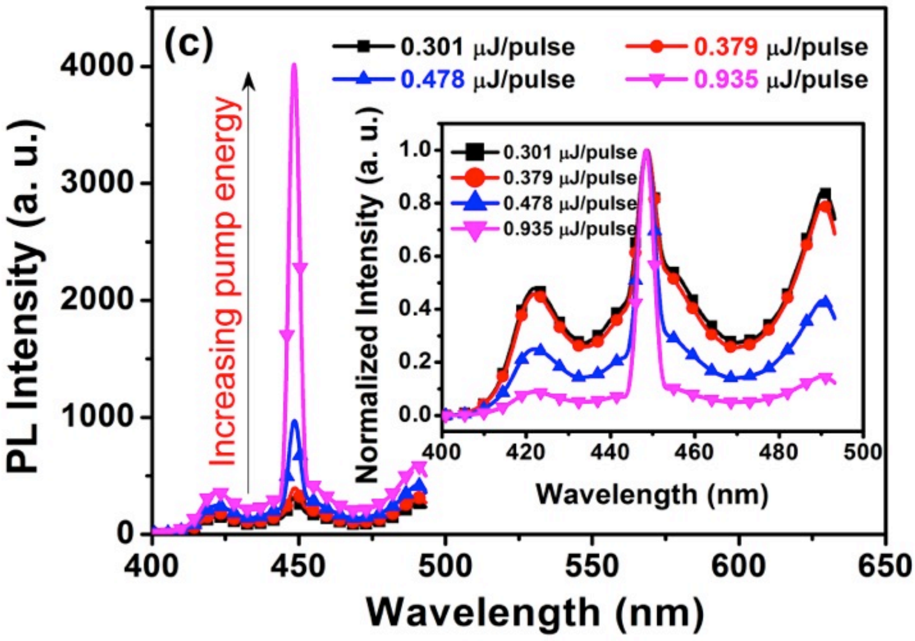

Figure S6. (a) The normalized output intensity versus the pump energy density at various thicknesses of the PEDOT:PSS layer for glass/ITO/PEDOT:PSS/PFO. (b) The output spectra of PFO at various pump energy for thickness of PEDOT:PSS layer at 23 nm. The ASE threshold obtained was 94.4 μJ/cm^2 (1.700 μJ/pulse) with the ASE peak at 447 nm and the FWHM of 3.9 nm. (c) The output spectra of PFO at various pump energy for glass/ITO/PPFN$^+$Br$^-$/PFO at optimized

PPFN$^+$Br$^-$ thickness of 37 nm. The corresponding ASE threshold was 26.6 mμJ/cm^2 (0.478 μJ/pulse) with the ASE peak at 448 nm and FWHM of 3.6 nm. The inset of (b) and (c) are the normalized spectra at each pump energy.

5. Refractive index measurement

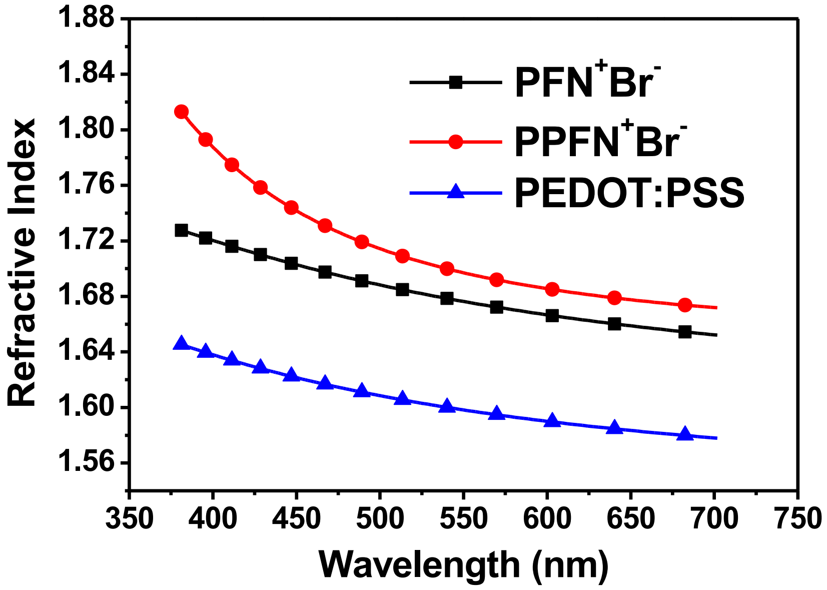

Figure S7. The refractive index of PFN$^+$Br$^-$, PPFN$^+$Br$^-$ and PEDOT:PSS.

6. ASE characterizations for device configuration of ITO/interlayer/PFO/interlayer

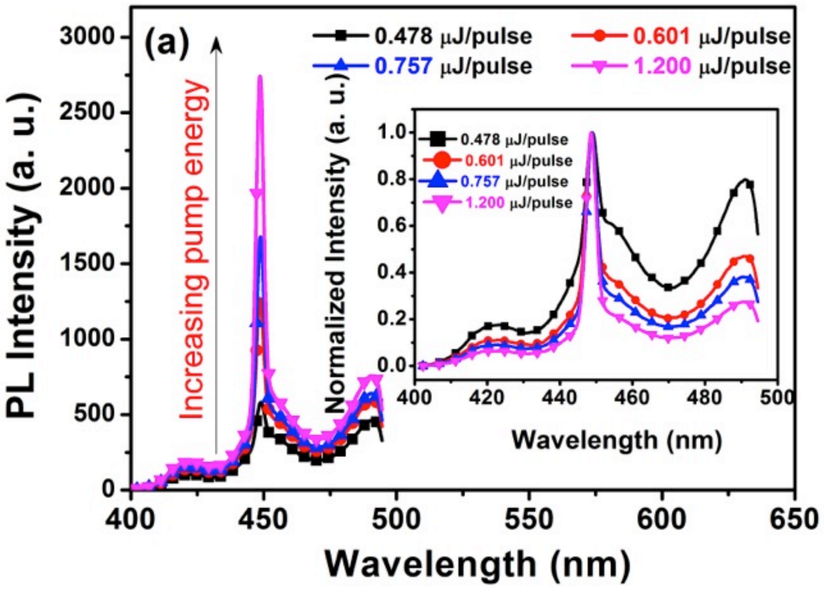

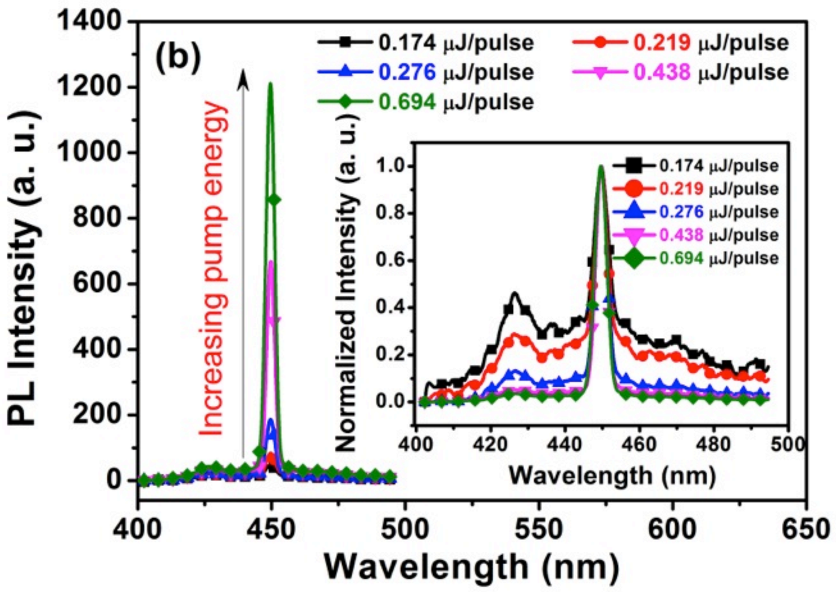

Figure S8. (a) The output spectra of PFO under various pump energy for Glass/ITO/PEDOT:PSS (23 nm)/PFO (90 nm)/PPFN$^+$Br$^-$ (17 nm). The ASE threshold, 33.4 μJ/cm^2 (0.601μJ/pulse) was obtained with the ASE peak at 449 nm and FWHM of 3.4 nm. (b) The output spectra of PFO under various pump energy for Glass/Ag (100 nm)/PPFN$^+$Br$^-$ (32 nm)/PFO (90 nm). The ASE threshold is 13.3 μJ/cm^2, namely 0.219 μJ/pulse with the peak at 449 nm and the FWHM of 3.5 nm. The insets of (a) and (b) show normalized spectra at each pump energy.

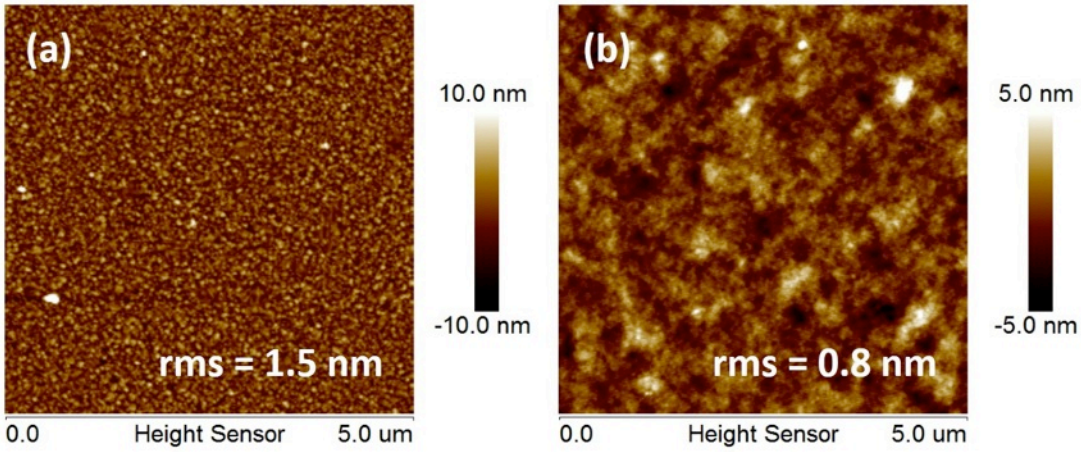

Figure S9 AFM images on surface morphology for (a) Ag electrode (40 nm) and (b) PFO (90 nm) on Ag (40 nm) electrode.

Reference

(1) Xu, W.; et al. A Hydrophilic Monodisperse Conjugated Starburst Macromolecule with Multidimensional Topology as Electron Transport/Injection Layer for Organic Electronics. *Polym. Chem.* **2014**, 5, 2942-2950.
